# Use of illicit substances and violent behaviour in psychotic disorders: two nationwide case-control studies and meta-analyses

**DOI:** 10.1017/S0033291719002125

**Published:** 2020-09

**Authors:** Jelle Lamsma, Wiepke Cahn, Seena Fazel, GROUP and NEDEN investigators

**Affiliations:** 1 Department of Psychiatry, University of Oxford, Oxford, UK; 2 Department of Psychiatry, University Medical Centre Utrecht, Utrecht, the Netherlands

**Keywords:** Aggression, drugs, psychosis, schizophrenia, violence

## Abstract

**Background:**

Substance use disorder explains much of the excess risk of violent behaviour in psychotic disorders. However, it is unclear to what extent the pharmacological properties and subthreshold use of illicit substances are associated with violence.

**Methods:**

Individuals with psychotic disorders were recruited for two nationwide projects: GROUP (*N* = 871) in the Netherlands and NEDEN (*N* = 921) in the United Kingdom. Substance use and violent behaviour were assessed with standardized instruments and multiple sources of information. First, we used logistic regression models to estimate the associations of daily and nondaily use with violence for cannabis, stimulants, depressants and hallucinogens in the GROUP and NEDEN samples separately. Adjustments were made for age, sex and educational level. We then combined the results in random-effects meta-analyses.

**Results:**

Daily use, compared with nondaily or no use, and nondaily use, compared with no use, increased the pooled odds of violence in people with psychotic disorders for all substance categories. The increases were significant for daily use of cannabis [pooled odds ratio (pOR) 1.6, 95% confidence interval (CI) 1.2–2.0), stimulants (pOR 2.8, 95% CI 1.7–4.5) and depressants (pOR 2.2, 95% CI 1.1–4.5), and nondaily use of stimulants (pOR 1.6, 95% CI 1.2–2.0) and hallucinogens (pOR 1.5, 95% CI 1.1–2.1). Daily use of hallucinogens, which could only be analysed in the NEDEN sample, significantly increased the risk of violence (adjusted odds ratio 3.3, 95% CI 1.2–9.3).

**Conclusions:**

Strategies to prevent violent behaviour in psychotic disorders should target any substance use.

## Introduction

Much of the excess risk of violent behaviour in psychotic disorders can be explained by substance use disorder (SUD) (Fazel *et al*., 2009). In a meta-analysis of 16 studies with a total of 5365 cases, SUD more than doubled the odds of violence [odds ratio (OR) 2.2, 95% confidence interval (CI) 1.6–2.9] (Witt *et al*., 2013).

However, it is unclear to what extent different categories of illicit substances, as defined by their psychopharmacological effects, are related to violent behaviour. Another uncertainty is whether subthreshold use, as opposed to SUD, is a risk factor for violence. These questions may be clinically relevant, as the psychopharmacological properties of substances modify violence risk in the general population (Tomlinson *et al*., 2016) and people with psychotic disorders are highly sensitive to the harmful effects of substances (Gregg *et al*., 2007). The few studies of cannabis (Koen *et al*., 2004; Moulin *et al*., 2018; Oluwoye *et al*., 2019) and stimulants (Bell *et al*., 2002; Miles *et al*., 2003; Harris *et al*., 2010) have produced conflicting results. Moreover, these studies have been limited by small samples of inpatients and proxy measures of violent behaviour (e.g. hostility, aggression). A recent meta-analysis of 12 studies involving 3873 subjects with severe mental illness – but not psychotic disorders specifically – reported a significant association between cannabis use and violence [pooled odds ratio (pOR) 3.0, 95% CI 2.0–4.5] (Dellazizzo *et al*., 2019). To our knowledge, there have been no studies of depressants (besides alcohol) or hallucinogens.

To address the limitations of previous studies, we have investigated the associations of daily and nondaily use with violent behaviour for cannabis, stimulants, depressants and hallucinogens in two nationwide samples of individuals with psychotic disorders.

## Methods

We used baseline data from two research projects: Genetic Risk and Outcome of Psychosis (GROUP) (Korver *et al*., 2012) and National Evaluation of the Development and Impact of Early Intervention Services (NEDEN) (Birchwood *et al*., 2014).

### Setting and participants

#### GROUP

GROUP is conducted by four university medical centres (i.e. Amsterdam Medical Centre, Maastricht University Medical Centre+, University Medical Centre Groningen, University Medical Centre Utrecht) and affiliated mental health centres (*k* = 36) in the Netherlands. These centres are located in geographically representative areas of the country and provide access to treatment in a variety of settings (e.g. psychiatric hospitals, outpatient clinics) to approximately 75% of the population. Recruitment took place in 2004. To be eligible for participation, patients had to (i) be aged between 16 and 50, (ii) have a good command of the Dutch language and (iii) meet Diagnostic and Statistical Manual of Mental Disorders, Fourth Edition, Text Revision (DSM-IV-TR) (American Psychiatric Association, 2000) criteria for schizophrenia or another psychotic disorder. In accordance with standard local practice, DSM-IV-TR diagnoses were made with the Comprehensive Assessment of Symptoms and History (Andreasen *et al*., 1992) or Schedules for Clinical Assessment for Neuropsychiatry (Wing *et al*., 1990).

#### NEDEN

All individuals enrolled into Early Intervention Services (EIS) at five sites across England (i.e. Birmingham, Cornwall, Cambridge, Norwich, Lancashire) between 2005 and 2009 were invited to participate. Sites were chosen to reflect urban and rural differences. The Department of Health and Social Care requires that people receiving EIS are between 14 and 35 years old and present with a first episode of psychosis. No additional inclusion criteria were set. The Operationalized Criteria System (McGuffin *et al*., 1991) was used to determine International Statistical Classification of Diseases and Related Health Problems, Tenth Edition (ICD-10) (World Health Organization [WHO], 1992) diagnoses of mental disorders.

### Measures

Information about the instruments' psychometric properties can be found in the relevant publications for GROUP (Korver *et al*., 2012) and NEDEN (Birchwood *et al*., 2014). Unless otherwise specified, the reference period was the lifetime.

#### GROUP

*Substance use*: The Substance Abuse Module of the Composite International Diagnostic Interview (CIDI-SAM) (WHO, 1990) was used to measure the frequency (i.e. daily use, nondaily use, no use) and severity (i.e. problematic use, nonproblematic use, no use) of substance use. The CIDI-SAM distinguishes between the following categories of substances: (i) alcohol; (ii) cannabis; (iii) cocaine; (iv) stimulants (e.g. amphetamine, khat); (v) sedatives (e.g. pentobarbital, diazepam); (vi) opiates (e.g. heroin, codeine); (vii) inhalants (e.g. toluene, butane); (viii) phencyclidine (PCP); (ix) psychedelics [e.g. lysergic acid diethylamide (LSD), mescaline]; and (x) other substances [e.g. amyl nitrite, 3,4-methylenedioxymethamphetamine (MDMA)]. Based on considerations of statistical power and similarities in psychopharmacological effects, we combined cocaine and stimulants as ‘stimulants’, sedatives, opiates and inhalants as ‘depressants’ and PCP and psychedelics as ‘hallucinogens’ (cf. Hill and Thomas, 2016). Cannabis, which has stimulant, depressive and hallucinogenic properties, was treated separately owing to the high prevalence of its use. We defined problematic alcohol use as an average intake of more than 18 standard drinks per week for men and more than 12 standard drinks per week for women during a minimum period of 2 weeks in the past year or 4 weeks at any other point in the past. These cutoffs reflect the median of several national guidelines and a consistent 1.5:1 male to female consumption ratio (Furtwaengler and de Visser, 2013). For other substances, problematic use corresponded to a DSM-IV-TR diagnosis of abuse or dependence.

*Violent behaviour*: Violent behaviour was established with the Life Chart Schedule (LCS) (Susser *et al*., 2000). Designed to record the development of symptoms, health care consumption and social functioning in schizophrenia patients, the LCS contains the following question regarding violence: ‘Did the patient physically attack or abuse someone else?’ The LCS was filled out based on review of clinical case notes and interviews with the patient and, if possible, one or both parents.

#### NEDEN

*Substance use*: A purposely designed questionnaire was used to assess substance use. For 15 substance categories, patients were asked whether they had used them: (i) almost every day; (ii) 1 to 3 times per week; (iii) less than once per week; (iv) 3 times or less; or (v) never. For the sake of consistency, we combined frequency categories ii, iii and iv as ‘nondaily use’ and refer to ‘almost every day’ as ‘daily’. The substance categories were rearranged as follows: (i) cannabis; (ii) stimulants (i.e. cocaine, amphetamine, khat); (iii) depressants (i.e. opiates, *γ*-hydroxybutyric acid, barbiturates, benzodiazepines, solvents, ‘poppers’); (iv) hallucinogens (i.e. LSD, psilocybin, ketamine); and (v) other substances (i.e. MDMA, ‘other’) (cf. Hill and Thomas, 2016).

*Violent behaviour*: Violent behaviour was ascertained from patient and clinician interviews using the Adverse Outcomes Questionnaire (AOQ). In the AOQ, a shortened version of the questionnaire used in the MacArthur Violence Risk Assessment Study (Steadman *et al*., 1998), violence is operationalized to encompass: (i) battery that resulted in physical injury; (ii) sexual assault; (iii) assault involving the use of a weapon; (iv) threats made with a weapon in hand; and (v) battery that did not result in physical injury. The AOQ referred to the past 12 months.

### Analyses

First, we used logistic regression models to estimate the associations of daily and nondaily use with violent behaviour for cannabis, stimulants, depressants and hallucinogens in the GROUP and NEDEN samples separately. Three comparisons were made: (i) daily use *v*. nondaily or no use; (ii) daily use *v*. no use; and (iii) nondaily use *v*. no use. For theoretical reasons, we included the confounders age, sex and educational level (Lamsma and Harte, 2015). Educational level, indicating whether a patient had completed secondary school, served as a proxy for socioeconomic status (Maksimović *et al*., 2008). We only analysed complete cases. Depending on the scale of measurement, complete and incomplete cases were compared on each model variable with the χ^2^-test (dichotomous) or *t* test (continuous). To improve validity, we required models with at least 5 observations per cell in the 2 × 2 table of the exposure and outcome of interest.

We then combined the results for the GROUP and NEDEN samples in random-effects meta-analyses. The *I*^2^ statistic was used as a measure of heterogeneity. Values of 25, 50 and 75% denoted low, moderate and high levels of heterogeneity, respectively (Higgins *et al*., 2003).

For sensitivity analyses, we examined severity of use. Alcohol, which has consistently been found to increase violence risk in people with psychotic disorders (Witt *et al*., 2013), was used as a positive control.

The level of statistical significance was set at 5%. Analyses were carried out in STATA 12.1.

## Results

Of the 1013 patients in the GROUP sample, 871 (86%) had data on all model variables and were thus included in the analyses. The corresponding numbers in the NEDEN sample were 1027 and 921 (90%), respectively. Complete cases differed significantly from incomplete cases on age [*t*_(1011)_ = 3.31, *p* = 0.001] in the GROUP sample (online Supplementary Table S1) and educational level [χ^2^ (1) = 3.89, *p* = 0.049] in the NEDEN sample (online Supplementary Table S2).

### Demographic and clinical characteristics of patients

#### GROUP

Demographic and clinical characteristics of the patients (*N* = 871) are presented in Table 1. The mean age was 27.3 years (s.d. = 7.1). Most patients were male (*n* = 673, 77%) and had received a diagnosis of schizophrenia (*n* = 602, 69%). Use of illicit substances was reported by 602 (69%) patients. About one if five patients had been violent (*n* = 179, 21%).

**Table 1.** Demographic and clinical characteristics of patients in the GROUP (*N* = 871) and NEDEN (*N* = 921) samples

	GROUP	NEDEN
Demographic characteristics		
Age, mean (s.d.) in years	27.3 (7.1)	22.8 (4.8)
Male	673 (77)	639 (69)
Caucasian	679 (79)	679 (74)
Completed secondary school	753 (86)	811 (88)
Clinical characteristics		
Psychiatric diagnosis^a^		
Schizophrenia	602 (69)	478 (47)
Schizoaffective disorder	106 (12)	70 (7)
Psychotic disorder NOS	74 (8)	190 (19)
Other	89 (10)	289 (28)
Age of onset, mean (s.d.) in years	23.1 (6.5)	21.3 (4.9)
Use of illicit substances		
Daily	430 (49)	314 (34)
Cannabis	411 (48)	285 (31)
Stimulants	68 (8)	55 (6)
Depressants	51 (6)	28 (3)
Hallucinogens	6 (1)	15 (2)
Other	23 (3)	21 (2)
Nondaily	427 (49)	480 (52)
Cannabis	170 (20)	279 (30)
Stimulants	198 (24)	245 (27)
Depressants	62 (8)	89 (10)
Hallucinogens	165 (20)	161 (17)
Other	212 (26)	230 (25)
Violent behaviour	179 (21)	204 (22)

s.d., standard deviation; NOS, not otherwise specified.

Data are *n* (%), unless otherwise stated.

a Psychiatric diagnoses were only available for the full NEDEN sample (*N* = 1027).

#### NEDEN

The patients (*N* = 921) had a mean age of 22.8 years (s.d. = 4.8) and were predominantly male (*n* = 639, 69%). (Table 1). The most common diagnosis was schizophrenia (*n* = 478, 47%). Almost two thirds of the patients had used illicit substances (*n* = 589, 64%). The prevalence of violent behaviour was 22% (*n* = 204).

### Primary analyses

#### GROUP

Daily use, compared with nondaily or no use, and nondaily use increased the adjusted odds of violent behaviour for all substance categories (Table 2). The increases were significant for daily use of stimulants [adjusted odds ratio (aOR) 2.2, 95% CI 1.3–3.8] and nondaily use of hallucinogens (aOR 1.8, 95% CI 1.2–2.7).

**Table 2.** Prevalence and risk of violent behaviour by different categories of illicit substances and frequency of their use in the GROUP (*N* = 871) and NEDEN (*N* = 921) samples

	*n* (%)			aOR (95% CI)^a^		
Substance category	DU	NDU	NU	DU *v*. NDU or NU	DU *v*. NU	NDU *v*. NU
GROUP						
Cannabis	99 (24)	36 (21)	44 (15)	1.4 (1.0–1.9)	1.5 (1.0–2.3)	1.3 (0.8–2.2)
Stimulants	25 (37)	48 (24)	98 (17)	2.2 (1.3–3.8)	2.6 (1.5–4.5)	1.5 (1.0–2.2)
Depressants	15 (29)	18 (29)	134 (19)	1.6 (0.9–3.1)	1.7 (0.9–3.3)	1.7 (0.9–3.0)
Hallucinogens	3 (50)	46 (28)	113 (17)	–	–	1.8 (1.2–2.7)
Other	8 (35)	57 (27)	100 (17)	1.9 (0.8–4.6)	2.4 (1.0–5.8)	1.7 (1.2–2.6)
NEDEN						
Cannabis	88 (30)	60 (21)	61 (17)	1.8 (1.3–2.5)	2.0 (1.3–2.9)	1.2 (0.8–1.8)
Stimulants	26 (46)	71 (28)	112 (17)	3.6 (2.1–6.4)	4.3 (2.4–7.8)	1.6 (1.2–2.3)
Depressants	13 (43)	23 (26)	173 (21)	3.3 (1.5–7.2)	3.4 (1.6–7.3)	1.2 (0.7–2.0)
Hallucinogens	7 (47)	44 (27)	158 (21)	3.3 (1.2–9.3)	3.4 (1.2–9.6)	1.3 (0.9–1.9)
Other	10 (45)	65 (27)	134 (19)	3.4 (1.4–8.2)	3.9 (1.6–9.3)	1.5 (1.0–2.1)

aOR, adjusted odds ratio; CI, confidence interval; DU, daily use; NDU, nondaily use; NU, no use.

Due to missing data, the total number of patients varies by substance category.

a Adjusted for age, sex and educational level.

#### NEDEN

When comparing daily use with nondaily or no use, cannabis (aOR 1.8, 95% CI 1.3–2.5), stimulants (aOR 3.6, 95% CI 2.1–6.4), depressants (aOR 3.3, 95% CI 1.5–7.2) and hallucinogens (aOR 3.3, 95% CI 1.2–9.3) significantly increased the adjusted odds of violence (Table 2). Nondaily use increased the aORs for these substance categories as well, with that for stimulants reaching statistical significance (aOR 1.6, 95% CI 1.2–2.3).

### Meta-analyses

Pooled across the GROUP and NEDEN samples, daily use of cannabis (pOR 1.6, 95% CI 1.2–2.0), stimulants (pOR 2.8, 95% CI 1.7–4.5) and depressants (pOR 2.2, 95% CI 1.1–4.5) significantly increased the odds of violence compared with nondaily or no use (Table 3). The same was found for nondaily use (compared with no use) of stimulants (pOR 1.6, 95% CI 1.2–2.0) and hallucinogens (pOR 1.5, 95% CI 1.1–2.1). Moderate heterogeneity was present for daily use of depressants in both comparisons (*I*^2^ = 46%, 50%). Otherwise, heterogeneity was low (*I*^2^ ⩽ 36%).

**Table 3.** Risk of violent behaviour by different categories of illicit substances and frequency of their use, pooled across the GROUP (*N* = 871) and NEDEN (*N* = 921) samples

	pOR (95% CI)		
Substance category	DU *v*. NDU or NU	DU *v*. NU	NDU *v*. NU
Cannabis	1.6 (1.2–2.0)	1.7 (1.3–2.3)	1.2 (0.9–1.7)
Stimulants	2.8 (1.7–4.5)	3.3 (2.0–5.4)	1.6 (1.2–2.0)
Depressants	2.2 (1.1–4.5)	2.3 (1.2–4.6)	1.4 (0.9–2.1)
Hallucinogens	–	–	1.5 (1.1–2.1)
Other	2.5 (1.4–4.7)	3.1 (1.6–5.7)	1.6 (1.2–2.1)

pOR, pooled odds ratio; CI, confidence interval; DU, daily use; NDU, nondaily use; NU, no use (using random-effects models).

### Sensitivity analyses

We observed no material differences in results after repeating the analyses in the GROUP sample with severity of use. As expected, alcohol increased the adjusted odds of violence (online Supplementary Table S3).

## Discussion

In two nationwide samples totalling 1792 individuals with psychotic disorders, we investigated associations between frequency of use and violence for different categories of illicit substances. Overall, daily and nondaily use of cannabis, stimulants, depressants, and hallucinogens were found to increase violence risk.

There are at least four ways in which substance use may lead to violent behaviour in psychotic disorders. First, psychopharmacological effects of intoxication with or withdrawal from substances (e.g. disinhibition, intensification of negative emotions) may lower the threshold for violence (Kuhns and Clodfelter, 2009). Substance use may also induce or exacerbate positive symptoms (e.g. delusions, hallucinations) (Winklbaur *et al*., 2006), which are risk factors for violent behaviour (Witt *et al*., 2013). This may be particularly relevant for cannabis and hallucinogens. The former has been found to increase the risk of developing a psychotic disorder (Di Forti *et al*., 2019), and the latter – with the possible exception of PCP – are thought not to increase violence risk in the general population (Tomlinson *et al*., 2016). Second, substance use may interfere with treatment. Individuals with problematic substance use are less likely to seek and adhere to treatment than those without these substance problems (Winklbaur *et al*., 2006). At the same time, substances may be used in an attempt to alleviate psychotic symptoms or unpleasant side effects of antipsychotics (Gregg *et al*., 2007). Self-medication increases the likelihood of avoidance or discontinuation of treatment and vice versa (Swartz *et al*., 1998). Substances may also reduce the therapeutic activity of antipsychotics (Lindsey *et al*., 2012). In the absence of effective treatment, positive symptoms may persist or worsen. Third, violence may occur during the commission of crimes to gain access to substances or the money to buy them (McGinty *et al*., 2016). Finally, users may become involved in illegal drug markets where violent behaviour is commonplace (Hodgins, 2008). Other explanations for the findings are confounding or mediation by biological (e.g. genetics, neurobiological abnormalities), psychological (e.g. cognitive impairment, personality pathology) or environmental (e.g. childhood maltreatment, erosion of social support) risk factors (Lamsma and Harte, 2015).

As far as we know, this is the largest study to investigate the relationship between use of illicit substances and violent behaviour in psychotic disorders. It has several strengths. First, the samples were drawn from diverse geographic areas and care settings. Sampling was also independent of the exposures and outcome of interest. This enhanced the generalizability of the results. Second, the use of multiple data sources increased the sensitivity of the LCS and AOQ as measures of violence. Finally, the findings for alcohol (as a positive control) were in the expected direction, supporting the validity of the design. However, there are several limitations. First, causality cannot be inferred, as the temporal relationship between substance use and violent behaviour was not known and we did not control for other confounders besides age, sex and educational level. Second, we included individuals who had used substances belonging to different categories, which may have biased risk estimates. Exclusion would have made cell counts too low for meaningful analyses of most substance categories. Third, daily use was a proxy measure of SUD. However, similar results were obtained for DSM-IV-TR diagnoses of abuse and dependence in the GROUP sample. Fourth, the definition and reference period for violence varied between GROUP and NEDEN. The more stringent definition and shorter reference period may explain why aORs were slightly higher in the NEDEN sample. Fifth, missing data may have limited the validity of the results. Sixth, the results of the meta-analyses should be treated with some caution: the estimation of the between-study variance, which is used in the calculation of the pooled effect size and its confidence interval, may be inaccurate when the number of studies is small (Borenstein *et al*., 2009). Seventh, we were unable to analyse PCP separately. Either too few patients had used PCP (GROUP) or no specific information was recorded for PCP (NEDEN). Finally, there has been a large increase in the use of novel psychoactive substances (NPS) in the years following data collection (Tracy *et al*., 2017). NPS are synthetic compounds designed to mimic the psychopharmacological effects of traditional substances (Miliano *et al*., 2016). Therefore, we hypothesize that NPS increase violence risk.

A clinical implication of the findings is that violence risk assessment in psychotic disorders should target any substance use. For structured instruments, it should be determined whether items for SUD and subthreshold use of different substance categories have incremental validity over a single item for SUD. The findings also suggest that interventions, which currently focus on SUD, may assist in the prevention of violent behaviour in patients with subthreshold use (Chang *et al.*, 2016). To clarify causal mechanisms, we recommend that studies further isolate the psychopharmacological effects of substances, use prospective designs and test for additional confounders and mediators.

## Supplementary material

For supplementary material accompanying this paper visit https://doi.org/10.1017/S0033291719002125. click here to view supplementary material
