# Supplementary material for: Use of illicit substances and violent behaviour in psychotic disorders: two nationwide case-control studies and meta-analyses
Source: Psychol Med. 2019 Aug 29;50(12):2028–33. doi: 10.1017/S0033291719002125 (PMC7525769; doi:10.1017/S0033291719002125)
Supplement: Supplementary file 1 [file S0033291719002125sup001.docx]

**SUPPLEMENTARY MATERIAL**

**GROUP investigators**

Therese van Amelsvoort^1^, Agna Bartels-Velthuis^2^, Richard Bruggeman^2,3^, Lieuwe de Haan^4,5^, Frederike Schirmbeck^4,5^, Claudia Simons^1,6^, Jim van Os^7,8^

^1^Department of Psychiatry and Neuropsychology, Maastricht, the Netherlands; ^2^University Centre for Psychiatry, University Medical Centre Groningen, Groningen, the Netherlands; ^3^Department of Clinical and Developmental Neuropsychology, University of Groningen, Groningen, the Netherlands; ^4^Department of Psychiatry, Academic Medical Centre, Amsterdam, the Netherlands; ^5^Arkin Institute for Mental Health, Amsterdam, the Netherlands; ^6^GGzE Institute for Mental Health Care, Eindhoven, the Netherlands; ^7^Department of Psychiatry, University Medical Centre Utrecht, Utrecht, the Netherlands; and ^8^Department of Psychosis Studies, King’s College London, London, UK

**NEDEN investigators**

Tim Amos^1^, Max Birchwood^2^, Linda Everard^3^, Nick Freemantle^4^

^1^Avon and Wiltshire Mental Health Partnership National Health Service Trust, Bristol, UK; ^2^Unit of Mental Health and Wellbeing, University of Warwick, UK; ^3^Birmingham and Solihull Mental Health National Health Service Foundation Trust, Birmingham, UK; and ^4^Department of Primary Care and Population Health, University College London, London, UK

**Table S1. Comparisons on model variables between complete and incomplete cases in the GROUP sample (*N* = 1013).**

Model variable CC (*n* = 871) IC (*n* = 142) Test statistic (*df*) *p*

Age, mean (SD) in years 27.3 (7.1) 25.2 (6.3) *t* (1011) = 3.31 .001

Male 673 (77) 117 (82) *χ^2^* (1) = 1.87 ns

Completed secondary school^a^ 753 (86) 106 (80) *χ^2^* (1) = 3.53 ns

Use of illicit substances 602 (69) 89 (75) *χ^2^* (1) = 1.60 ns

Violent behaviour 179 (21) 4 (20) - -

CC, complete cases; IC, incomplete cases; *df*, degrees of freedom; SD, standard deviation; ns, nonsignificant.

Data are *n* (%), unless otherwise stated. Due to missing data, the number of incomplete cases varies by model variable.

**Table S2. Comparisons on model variables between complete and incomplete cases in the NEDEN sample (*N* = 1027).**

Model variable CC (*n* = 921) IC (*n* = 106) Test statistic (*df*) *p*

Age, mean (SD) in years 22.8 (4.8) 22.1 (5.3) *t* (1008) = 1.35 ns

Male 639 (69) 70 (66) *χ^2^* (1) = 0.50 ns

Completed secondary school^a^ 811 (88) 61 (80) *χ^2^* (1) = 3.89 .049

Use of illicit substances 589 (64) 46 (67) *χ^2^* (1) = 0.21 ns

Violent behaviour 204 (22) 15 (23) *χ^2^* (1) = 0.03 ns

CC, complete cases; IC, incomplete cases; *df*, degrees of freedom; SD, standard deviation; ns, nonsignificant.

Data are *n* (%), unless otherwise stated. Due to missing data, the number of incomplete cases varies by model variable.

**Table S3. Prevalence and risk of violent behaviour by severity of use of alcohol^a^ and different categories of illicit substances in the GROUP sample (*N* = 871).**

*n* (%) aOR (95% CI)^b^

Substance category PU NPU NU PU *vs* NPU or NU PU *vs* NU NPU *vs* NU

Alcohol 83 (24) 74 (19) 21 (16) 1.5 (1.1-2.1) 1.8 (1.0-3.1) 1.3 (0.7-2.2)

Cannabis 90 (24) 45 (21) 44 (15) 1.3 (0.9-1.9) 1.5 (1.0-2.4) 1.4 (0.8-2.2)

Stimulants 43 (30) 31 (23) 98 (17) 1.8 (1.2-2.7) 2.0 (1.3-3.0) 1.4 (0.9-2.3)

Depressants 15 (33) 18 (24) 134 (19) 1.9 (1.0-3.7) 2.0 (1.0-3.9) 1.3 (0.7-2.4)

Hallucinogens 12 (27) 41 (30) 113 (17) 1.3 (0.7-2.7) 1.6 (0.8-3.2) 2.0 (1.3-3.1)

Other 28 (29) 42 (28) 100 (17) 1.5 (0.9-2.4) 1.9 (1.1-3.1) 1.9 (1.2-2.9)

aOR, adjusted odds ratio; CI, confidence interval; PU, problematic use; NPU, nonproblematic use; NU, no use.

Due to missing data, the total number of patients varies by substance category.

^a^Alcohol was used as a positive control.

^b^Adjusted for age, sex and educational level.
